# Biological Effects of *Rosaceae* Species in Skin Disorders—An Up-To-Date Overview

**DOI:** 10.3390/plants14111605

**Published:** 2025-05-24

**Authors:** Andreea Maria Cristea, Andreea Smeu, Ioan-Alexandru Cîmpeanu, Andrada Iftode, Sergio Liga, Diana-Simona Tchiakpe-Antal, Daliborca Vlad, Cristina Adriana Dehelean, Dan Iliescu

**Affiliations:** 1 University Clinic of Toxicology, Drug Industry, Management and Legislation, Faculty of Pharmacy, “Victor Babes” University of Medicine and Pharmacy, 2nd Eftimie Murgu Square, 300041 Timisoara, Romania; andreea.cristea@umft.ro (A.M.C.); andreea.geamantan@umft.ro (A.S.); andradaiftode@umft.ro (A.I.); cadehelean@umft.ro (C.A.D.); 2 Research Centre for Pharmaco-Toxicological Evaluation, Faculty of Pharmacy, “Victor Babes” University of Medicine and Pharmacy, 2nd Eftimie Murgu Square, 300041 Timisoara, Romania; diana.antal@umft.ro; 3 Doctoral School, “Victor Babes” University of Medicine and Pharmacy, Eftimie Murgu Square 2, 300041 Timisoara, Romania; 4 Department of Applied Chemistry and Engineering of Organic and Natural Compounds, Faculty of Chemical Engineering, Biotechnologies and Environmental Protection, Politehnica University Timisoara, Vasile Pârvan No. 6, 300223 Timisoara, Romania; sergio.liga96@gmail.com; 5 Department of Pharmaceutical Botany, Faculty of Pharmacy, “Victor Babes” University of Medicine and Pharmacy, 2nd Eftimie Murgu Square, 300041 Timisoara, Romania; 6 Biochemistry and Pharmacology Department, Discipline of Pharmacology, “Victor Babes” University of Medicine and Pharmacy, 300041 Timisoara, Romania; vlad.daliborca@umft.ro; 7 University Clinic of Surgical Semiology I and Thoracic Surgery, Faculty of Medicine, “Victor Babes” University of Timisoara, 2 Eftimie Murgu Square, 300041 Timisoara, Romania; dan.iliescu@umft.ro

**Keywords:** *Rosaceae* family, phytotherapy, *Eriobotrya japonica*, skin disorders, biological effects

## Abstract

The *Rosaceae* family, comprising over 3000 species, has been extensively investigated for its therapeutic potential, particularly in dermatological applications. Skin illnesses have become in recent years a serious burden worldwide, with more than 3 billion individuals of all ages affected by a skin condition. This review focuses on approximately 50 species from genera such as *Rosa*, *Rubus*, *Prunus*, *Potentilla*, and especially *Eriobotrya japonica*, which have shown promising biological effects due to their diverse bioactive compounds. This review provides a current perspective on the recent scientific literature that highlights the role of *Rosaceae* members in managing various skin disorders. Key dermatological conditions addressed include dermatitis, acne, skin aging, melanoma, and psoriasis. By summarizing both in vitro and in vivo findings, this review underscores the importance of *Rosaceae* species in the development of plant-based dermatological therapies and encourages further research into their mechanisms of action and clinical potential.

## 1. Introduction

Skin illnesses have become in recent years an issue of public health concern and a serious burden worldwide [1]. According to recent data, more than 3 billion individuals of all ages are affected by a skin condition. These diseases are challenging due to their multilevel impact (physical and mental health and social status), leading to poor quality of life [2]. Skin disorders are a heterogeneous group that encompasses chronic diseases, such as psoriasis and atopic dermatitis, skin cancers (fatal—melanoma —and non-fatal), and even rare illnesses, such as genodermatoses [1]. At present, the existing therapeutic approaches offer symptomatic relief or act only as partial cures. In addition, the most frequently recommended treatments are invasive and exert multiple adverse effects (topical and systemic therapies, surgery, radiation, immunotherapy, chemotherapy) [3]. In light of these inconveniences, an increased interest has been recorded on the use of natural products as potential treatments for skin disorders.

Thus, in dermatology, phytotherapy has gained renewed attention due to its efficacy, affordability, and the advantageous safety profile of most phytocompounds compared to synthetic medicines [4]. Among the wide variety of plants studied for their potential in managing skin diseases are species belonging to the *Rosaceae* family, which stand out for their diversity and therapeutic properties. Among the most pharmacologically valuable botanical families, the *Rosaceae* family encompasses some 3500 species (mostly distributed in temperate zones) and includes ornamental, medicinal, and fruiting plants [5,6]. Beyond their horticultural and economic importance, these species are known for their richness in phenolic acids, flavonoids, anthocyanins, flavonols, and ellagitannins [7].

This phytochemical profile provides the species from the *Rosaceae* family with potent anti-inflammatory and antioxidant effects, highlighting their potential to address various skin-related conditions. For example, *Rosa canina* is notable for its high concentrations of vitamin C and phenolic compounds, making it a good candidate for wound healing and promoting skin quality [8]. Similarly, species such as *Fragaria vesca* (wild strawberry) and *Malus domestica* (apple) are rich in phenolic acids and flavonoids, which have significant potential in scavenging free radicals and stimulating skin regeneration [9]. Another example is *Eriobotrya japonica* (loquat), which is a significant source of triterpenoids and phenolic compounds, making it valuable in therapeutic approaches for acne, psoriasis, skin hyperpigmentation, and inflammatory skin disorders, as well as showing promising potential in cancer treatment [10,11]. Other members of the *Rosaceae* family, such as *Cydonia oblonga, Prunus persica*, and *Rosa alba,* have been highlighted in various reviews for their beneficial effects on skin conditions, including chapped skin, itching, burns, allergies, hyperpigmentation, and rosacea [12].

Given the aforementioned points, this review prioritizes recent scientific advances in the actions of about 50 *Rosaceae* species in different skin-related diseases, with most included studies published in the last five years, to provide the state of the art and updated findings on the implications of *Rosaceae* species. This review highlights their potential in skin health, offering insight into their rich phytochemical profiles, therapeutic effects, biological activities, and mechanisms of action, supporting the knowledge of these effects by providing in vitro and in vivo studies.

**Figure 1:**
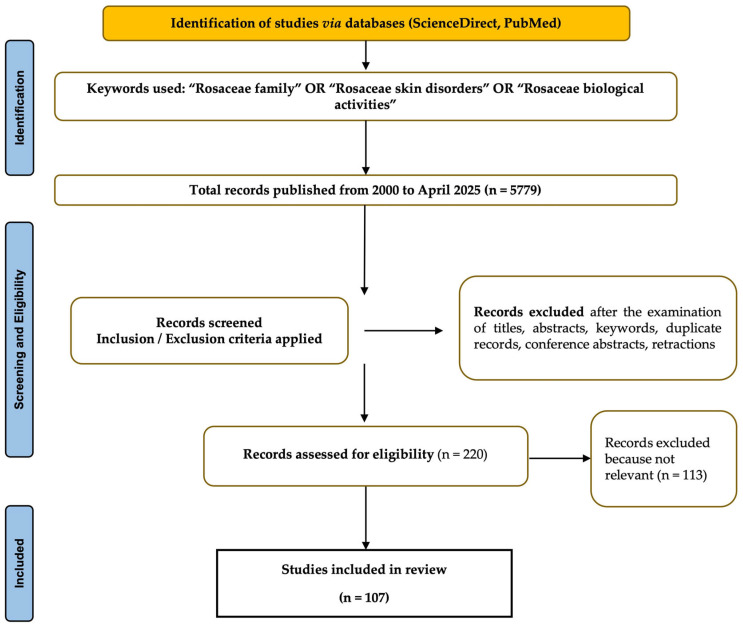
PRISMA flow diagram of the bibliographic research. (*n* = number of records.)

To the best of our knowledge, the present review is the first systematic review evaluating *Rosaceae* species for potential dermatological applications, aiming to analyze datasets that have been published up to April 2025 in peer-reviewed journals and databases, including PubMed and ScienceDirect, by combining the following keywords: *Rosaceae* family, *Rosaceae* skin diseases, *Rosaceae* species, and *Rosaceae* biological activities. The focus was placed on retrieving open-access literature and non-open-access sources based on scientific relevance and methodological rigor. Most included study articles (approximately 56%) were published within the last five years, 24% were published within the previous 10 years, and 20% were published more than 10 years ago, emphasizing recent scientific advances. To summarize the methodology followed, Figure 1 illustrates the PRISMA literature search flow diagram.

## 2. General Aspects of Skin Disorders

Inflammation is a natural and essential response of the skin to injury and infection, but the intensity and duration of this response are critical to prevent damage to skin tissues. If inflammation is not treated correctly and quickly, it can lead to dysregulation of skin homeostasis and the development of chronic inflammatory dermatoses such as atopic dermatitis or psoriasis [13]. The development of atopic dermatitis can be influenced by numerous factors such as immune disorders, skin barrier dysfunction, and itching. Most studies report an imbalance of T helper type 1 (Th1) and Th2 T helper cells [14]. The predominant Th2-type immune response includes the production of proinflammatory cytokines, such as IL-4, IL-5, and IL-13, which stimulate the production of IgE (Immunoglobulin E) antibodies and attract eosinophils to affected areas. IgE binds to FcεRI receptors on dendritic cells and mast cells, leading to the release of histamines and other inflammatory substances [15]. Psoriasis is also triggered when genetic or environmental factors activate plasmacytoid dendritic cells, leading to the production of numerous proinflammatory cytokines, including tumor necrosis factor (TNF)-α, interferon (IFN)-γ, and interleukins. Many of these cytokines stimulate keratinocyte hyperproliferation, which perpetuates a cycle of chronic inflammation [16]. Although psoriasis is a chronic inflammatory disease, some studies evaluated the efficacy and safety of herbal preparations that can be used topically for psoriasis [17]. Also, examples of species that were evaluated as a topical formulation and demonstrated antipsoriasis properties and low cytotoxicity are *Rosa damascena* and *Rosa canina* [18].

Exposure to ultraviolet (UV) radiation, in particular UV-B, is the main environmental risk factor for the development of melanoma, a pathology whose incidence has been increasing rapidly in recent times [19]. Melanomas are associated with a large number of somatic mutations, particularly in genes that control cell proliferation, metabolism, and survival, such as BRAF, NRAS, PTEN, and CDKN2A. The most common genetic abnormalities are BRAF mutations, which activate the MAPK pathway, responsible for cell cycle dysregulation and inhibition of apoptosis [20]. Numerous herbal phytoconstituents have demonstrated anticarcinogenic effects, including inhibition of cell proliferation, induction of apoptosis (programmed cell death), blocking metastasis, and prevention of angiogenesis [21].

Furthermore, exposure to UVB radiation can lead to oxidative stress, which generates reactive oxygen species (ROS) that can damage skin cells and induce inflammation. This damage is commonly associated with the photoaging process, characterized by wrinkles, loss of elasticity, and decreased skin hydration, mainly caused by the activation of inflammatory transcription factors and collagen-degrading enzymes [22].

Another important aspect may be the excessive production or uneven distribution of melanin, leading to various dermatologic disorders such as hyperpigmentation, lentigines, age spots, melasma, and others. It may be associated with conditions such as post-inflammatory melanosis and changes in the immune system of the skin. Thus, regulation of melanin synthesis is essential in preventing these conditions. Tyrosinase (TYR) is the main factor responsible for melanin biosynthesis, and inhibiting it can reduce melanin production and may be an effective strategy for skin depigmentation. Many chemical agents, such as hydroquinone, azelaic acid, and kojic acid, are used to inhibit tyrosinase activity. However, these agents have limitations in terms of skin penetration, low stability, and side effects [23].

An overview of the effective medicinal plants of the *Rosaceae* family is presented in this study, emphasizing both the bioactive components and the potential beneficial actions on the skin.

## 3. General Aspects of the *Rosaceae* Family

Based on the APG IV criteria (Angiosperm Phylogeny Group), the *Rosaceae* family is classified as part of the Rosales order, class Magnoliopsida, alongside families such as *Moraceae*, *Rhamnaceae*, *Cannabaceae*, *Ulmaceae*, and *Urticaceae*, which all constitute a monophyletic eudicots group [24,25].

**Figure 2:**
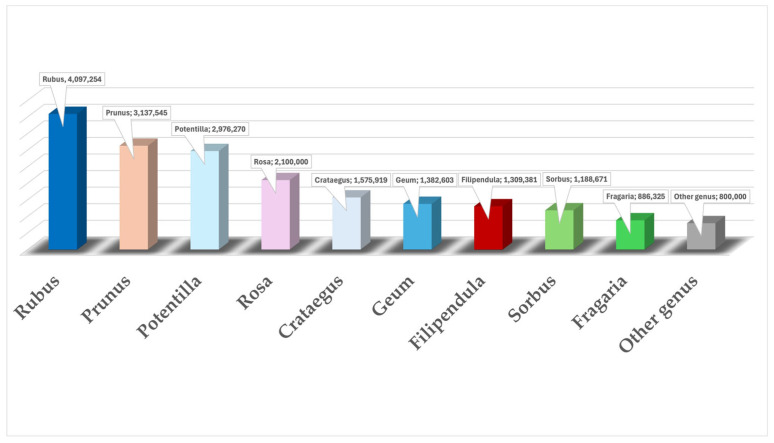
Taxonomic distribution of genus occurrences from the *Rosaceae* family [https://www.gbif.org/species/5015/metrics] (accessed on 28 February 2025).

The *Rosaceae* family is a group of flowering plants that encompasses approximately 105 genera and is mostly found in temperate and subtropical regions. Herbaceous plants (*Fragaria* and *Potentilla* genus), shrubs (*Rubus* and *Rosa* genus), and trees (*Prunus* genus) are the members of the group, and they have significant ecological, food, and therapeutic characteristics [26]. *Rosaceae* family’s members share several key botanical morphological features despite their diversity, such as the following: (i) typically fibrous or taproot systems, with mycorrhizal fungi; (ii) leaves that are mostly alternate, pinnate, or palmate, with the presence of stipules; (iii) solitary flowers, racemose inflorescences, or panicles depending on the species; (iv) actinomorphic pentamerous floral structure; (v) highly diverse fruit types (e.g., drupes, pome, aggregate fruits, capsules); and (vi) entomophilous pollination [27,28,29,30]. There are now only three subfamily divisions compared to the previous four, which consisted of *Amygdaloideae*, *Maloideae*, *Rosoideae*, and *Spiraeoideae*. The classification of three new subfamilies, (i) *Amygdaloideae* (including *Maloideae* and *Spiraeoideae*), (ii) *Dryadoideae*, and (iii) *Rosoideae*, was achieved with the help of phylogenetic analysis on six nuclear and four chloroplast genes [31,32]. The taxonomic distribution of genus occurrences in the *Rosaceae* family is depicted in Figure 2.

## 4. Phytochemical Composition of the *Rosaceae* Family

**Figure 3:**
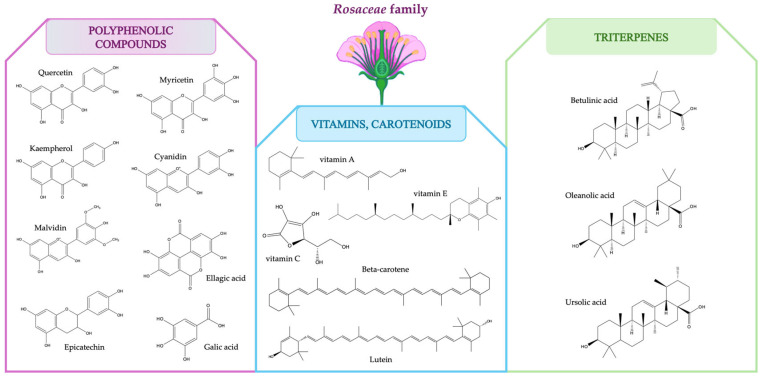
Most prevalent bioactive molecules found in the *Rosaceae* family.

The therapeutic benefits of plants in the *Rosaceae* family and other medicinal species have been widely acknowledged, particularly in the areas of skincare, inflammation regulation, and overall health maintenance [33]. Polyphenolic compounds are receiving a considerable amount of attention due to their diverse bioactivities [34]. *Rosaceae* plants’ health benefits are largely due to the presence of flavonoids, anthocyanins, procyanidins, and tannins, among others (e.g., polysaccharides, vitamins, carotenoids). In addition to polyphenolic compounds, there is a distinct group of bioactive compounds that encompasses triterpenes and volatile oils. Figure 3 shows the bioactive molecules that are most prevalent in the *Rosaceae* family.

### 4.1. Polyphenolic Compounds (Flavonoids, Anthocyanins, and Hydrolyzable Tannins)

Cosmetology is becoming more aware of the beneficial effects of polyphenols on skin health, which are known for their wide range of biological activities. Polyphenols have become known as effective antiaging agents because of their properties, which result in improved skin elasticity, reduced wrinkles, and overall healthier, more youthful-looking skin. The synthesis of collagen and elastin is stimulated by these compounds, which promote skin cell renewal and eliminate oxidative stress [7,35,36,37]. The broad spectrum of biological activities of phenolic compounds is mainly due to their antioxidant, anti-inflammatory, antimicrobial, and anticancer properties [34,38,39,40,41,42]. For example, the flavonoids, such as quercetin, kaempferol, and myricetin, widely found in the *Rosaceae* family (e.g., apples, cherries, strawberries, and roses) exhibit powerful antioxidant, anti-inflammatory, antiaging, and photoprotective properties, making them valuable for skin health and dermatological applications [43,44,45]. Their bioactive properties make them essential in modern skincare formulations for UV protection, acne treatment, and wrinkle reduction. Another example, the most common flavan-3-ols, including catechins, epicatechins, and procyanidins, play a crucial role in maintaining skin hydration, elasticity, and protection from environmental damage [46,47,48]. Also, anthocyanins’ usefulness in modern skincare products and natural remedies is due to their ability to reduce wrinkles, brighten skin, and protect collagen [49,50,51,52]. Hydrolyzable tannins include ellagitannins and gallotannins, which break down into ellagic acid and gallic acid, respectively. These compounds provide potent antioxidant, anti-inflammatory, antiaging, and antimicrobial benefits for the skin [53,54].

### 4.2. Volatile Oils

The *Rosaceae* family (e.g., roses, apples, cherries, raspberries, strawberries) is rich in triterpenes and volatile oils that have antiaging, anti-inflammatory, wound-healing, and antimicrobial benefits for the skin. The use of these bioactive compounds in skincare formulations is widespread for moisturization, regeneration, and protecting against environmental stressors [55,56,57,58]. The presence of volatile oils, especially from *Rosa* species, provides a valuable contribution in protecting the skin against oxidative stress while helping to reduce inflammation and regulate pigmentation processes. These oils (geraniol, citronellol, or methyl-eugenol) may act synergistically with other plant compounds to promote skin health and balance, including in the context of inflammatory skin conditions [59,60]. Another example is *Rosa alba* essential oil, which predominantly comprises volatile oils (e.g., citronellol, geraniol, nerol) and shows strong antimicrobial activity, with superior efficacy against Gram-positive bacteria (*Staphylococcus* and *Bacillus* spp.), while Gram-negative strains, especially *Pseudomonas* spp., display marked resistance; in addition, the essential oil showed significant antifungal activity against *Aspergillus flavus* and *Aspergillus niger* [12].

### 4.3. Triterpenes, Tetraterpenes (Carotenoids), and Vitamins

*Rosaceae* family plants are known for their prominent triterpenoids, which include ursolic acid, oleanolic acid, and betulinic acid. These acids have antiaging, anti-inflammatory, antioxidant, wound-healing, and antimicrobial properties, which make them crucial for skincare formulations [61,62,63,64,65,66]. Vitamins and carotenoids play a crucial role in skin protection, repair, and rejuvenation [67,68,69,70,71]. Among vitamins and carotenoids, the ones used in cosmetics most often are vitamin E, vitamin C, vitamin B complex (vitamin B3, B5, and B7), β-carotene, lutein, zeaxanthin, and lycopene [72].

## 5. Benefits of Natural Products from *Rosaceae* Plants Targeting the Skin

**Figure 4:**
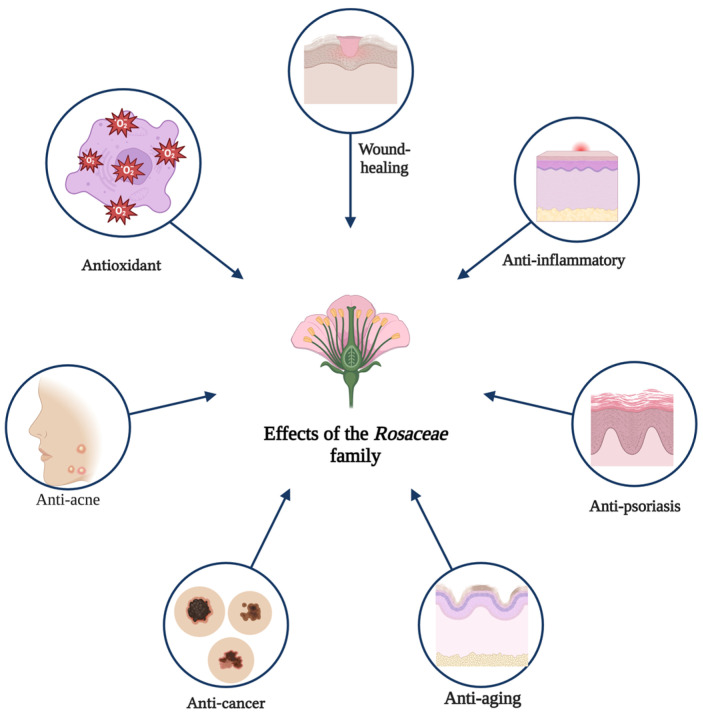
Biological activities of *Rosaceae* members regarding skin disorders.

Due to the diversity of biomolecules with multiple therapeutic benefits, plants of the *Rosaceae* family have been the subject of intense investigation. Nowadays, their potential has also been demonstrated in the field of cosmetology, with a growing interest in developing products based on natural ingredients [73].The figure below (Figure 4) shows the effects for which these plants are intensively studied and which contribute to the discovery of their therapeutic potential.

### 5.1. Anti-Inflammatory Activity

*Rosaceae* species have been intensively studied for their anti-inflammatory properties [74]. *Eriobotrya japonica* L., a species of fruit tree known as loquat, with an original habitat in China and Japan, is recognized for its benefits in addressing some dermatological conditions, with anticarcinogenic, antioxidant, and anti-inflammatory effects [75]. In addition, the anti-inflammatory effect can be attributed to the potential of extracts from this species in modulating key proinflammatory pathways, such as Mitogen-Activated Protein Kinase P38 (p38 MAPK), Extracellular Signal-Regulated Kinase (ERK), and Nuclear Factor Kappa B (NF-κB) [76]. In this regard, the anti-inflammatory activity of a methanolic leaf extract of loquat was evaluated in vivo by Norihiro et al. The results indicated the inhibitory effect of triterpenes, such as oleanane, ursane, and lupane derivatives, on mice’s 12-O-tetradecanoylphorbol-13-acetate-induced inflammation and ear edema. Specifically, all the tested derivatives demonstrated an anti-inflammatory effect at a 50% inhibitory dose ranging between 0.03 and 0.43 mg/ear [77]. Similarly, *Filipendula palmata* (Siberian meadowsweet), a species originally native to the northern part of China, is used in folk medicine for digestive disorders, rheumatism, epilepsy, and after skin burns while also presenting anti-inflammatory and antioxidant effects and benefits in gynecology [78]. In this context, the in vitro anti-inflammatory effect of a 70% ethanolic extract at a concentration of 100 μg/mL of *Filipendula palmata* was assessed by Xiao et al. on human epidermal keratinocytes (HaCaT cell line). Analogous to *Eriobotrya japonica*, *Filipendula palmata* also modulates NF-κB and MAPK signaling, confirming a recurrent anti-inflammatory pattern in *Rosaceae* species [79]. *Cydonia oblonga* Mill. (quince), native to West Asia, exhibits beneficial health effects, including anticarcinogenic, anti-inflammatory, and antimicrobial properties [80]. The anti-inflammatory effect is associated with inhibition of the NF-κB and p38MAPK pathways, as well as activation of the AKT pathway [81], which plays a pivotal role in resolving inflammation. Herrera-Rocha et al. demonstrated that apos-acetonic extract (85%) from the fruits of *Cydonia oblonga* species significantly inhibits COX-2 enzyme (52.31 ± 0.01%), an effect attributed to phenolic compounds such as hydroxycinnamic acids, flavanols, and flavanones [82]. Another species with notable anti-inflammatory activity is *Pyrus ussuriensis* Maxim (Korean pear), commonly used to treat diseases such as atopic dermatitis in China and Korea [83]. Cho et al. evaluated, both in vitro and in vivo, the benefits of *Pyrus ussuriensis* extract in terms of a potential therapeutic effect on atopic dermatitis. The findings revealed that astragalin (the main compound of the chloroform-soluble fraction of leaves from this species) decreased proinflammatory markers, such as nitric oxide in mouse macrophages (RAW 264.7 cells) and IL-6 and IL-1β in tumor necrosis factor (TNF-α)/interferon γ (IFNγ)-stimulated HaCaT cells, significantly reducing atopic dermatitis in animal models [84]. *Crataegus* spp. (Hawthorn), a genus of the *Rosaceae* family comprising around 280 species, is widely recognized for its medicinal properties and classified by the European Medicines Agency as a traditional herbal medicinal product. Extracts from species such as *Crataegus monogyna*, *C. laevigata*, *C. mexicana*, and *C. douglasii* exhibit anti-inflammatory activity, primarily through modulation of cytokine production. Specifically, C-glycosyl flavones in these extracts downregulate proinflammatory mediators (TNF-α, IL-1β, IL-6, IL-33, nitric oxide, and prostaglandin E₂) while promoting the anti-inflammatory cytokine IL-10 [85,86].

Another species to consider is *Rubus idaeus* L. (raspberry), a widely distributed plant in Europe, especially in Russia and Poland, and cultivated in countries such as the United States and Mexico. Known for its high polyphenolic content, including flavonoids, anthocyanins, and ellagitannins, *Rubus idaeus* L. is recognized for its anti-inflammatory properties, primarily by inhibiting lipoxygenase and cyclooxygenase [87]. *Rosa damascena* Mill. (Damask rose), native to Europe and the Middle East, is another noteworthy species. Apart from its wide use in the cosmetic and fragrance industries, *Rosa damascena* L. exhibits numerous medicinal properties, such as analgesic effects, benefits for the digestive tract and cardiovascular system, and anti-inflammatory effects through the suppression of gene expression for inflammation biomarkers [88]. Also, extracts from *Cotoneaster hissaricus* and *Cotoneaster hsingshangensis* showed promising effects for skin health by protecting against oxidative stress and inflammation and inhibiting hyaluronidase, an enzyme implicated in hyaluronic acid degradation. The ethyl acetate fraction from *C. hsingshangensis* was the most effective in inhibiting hyaluronidase, having a lower IC50 than standard EGCG, suggesting significant potential for preventing dermal matrix damage. In addition, extracts from *Cotoneaster* leaves inhibited COX-1 and COX-2 cyclooxygenases, enzymes essential in the inflammatory process. These effects suggest a significant anti-inflammatory potential of *Cotoneaster*, with applications in dermatologic and cosmetology treatments, especially for the management of inflammation and oxidative stress affecting the skin [89].

In the context of cutaneous inflammation, extracts from *Cotoneaster roseus* and *Cotoneaster nebrodensis* also showed a particular therapeutic potential in the treatment of acne by suppressing key enzymes involved in inflammatory processes [90]. According to a study by Krzemińska B. et al., methanol–acetone–water extract of *C. roseus* fruits was effective against hyaluronidase and lipoxygenase, and both species showed significant inhibition of COX-1 and COX-2, supporting their potential as natural anti-inflammatory agents with applications in conditions such as acne [91].

A chronic inflammatory skin condition characterized by pruritic and eczematous lesions could be atopic dermatitis [92], and a *Rosa multiflora* root extract and RM-3 (a type of condensed tannin) were evaluated for the treatment of atopic dermatitis-like skin lesions induced by mites in NC/Nga mouse models. Both demonstrated significant effects in relieving atopic dermatitis symptoms and reducing signs of inflammation and pruritus. *Rosa multiflora* root extract and RM-3 reduced the levels of iNOS and COX-2 (molecules involved in inflammation), thus demonstrating an effective anti-inflammatory effect, which could be useful for the symptomatic treatment of dermatitis [93].

A summary table showing species of the *Rosaceae* family, their main mechanism of action, and the issue on which they act is presented below (Table 1).

### 5.2. Wound-Healing Effects

An increasing number of research studies are investigating the various therapeutic potentials of herbal medicine. Certain species from the *Rosaceae* family have demonstrated a significant impact on wound healing [94]. For instance, in an experimental animal model, the aqueous extract of *Cydonia oblonga* Mill. exhibited a notable capacity to promote skin lesion repair [95]. Another example is from the in vitro study conducted by Van del Velde et al. that evaluated the effect of *Fragaria ananassa* (strawberry) and *Rubus* species (blackberry) extracts on skin fibroblast migration in HDFa fibroblasts, both of which are rich in polyphenolic compounds. The findings indicate that their anthocyanin-enriched fractions of crude extract exhibited an increase in fibroblast migration (50% compared to the control) at a concentration of 1 μg/mL, revealing their potential to promote wound healing [96]. Another species that could be mentioned is *Prunus africana* (Pygeum or African cherry), which is an evergreen species. Traditionally, it is used for its potential in addressing ailments such as hypertension, respiratory tract, and digestive disorders and for its properties in accelerating skin lesion repair. The in vivo assessment on an experimental animal model using 5% (*w*/*w*) and 10% (*w*/*w*) crude methanolic extract ointments conducted by Hanbisa et al. suggested that the species possesses a significant wound-healing effect [97]. *Alchemilla vulgaris* (lady’s mantle) is also recognized for its benefits in dermatological conditions such as eczema, ulcers, and dermatitis. It also exhibits wound-healing properties. In this sense, a study showed that the healing effect can be attributed to the promutagenic effect on epithelial cells [98].

*Pourthiaea villosa* extract demonstrates inhibition of the secretion of collagenolytic MMPs (MMP-2, MMP-9, and MMP-3) and oxidative stress (H2O2)-induced collagen degradation in human dermal fibroblasts. Treatment with the extract protects the collagen matrix of the skin by reducing the activity of MMPs and downregulating the MAPK signaling pathway, demonstrating a protective effect against skin aging and collagen degradation [99]. Collagen protection is, therefore, essential for the wound-healing process, as collagen not only provides structural support but also regulates biological processes, including inflammation and angiogenesis. The controlled degradation of collagen by specific proteases, such as matrix metalloproteinases, facilitates tissue regeneration, while an imbalance in this process can lead to pathological conditions such as fibrosis or poor wound healing [100].

Findings from the specialized literature have shown that different species from the *Rosaceae* family could provide a significant impact in terms of wound repair and healing, being a promising therapeutic alternative, demonstrating the ability to protect the cutaneous organ and stimulate collagen [95,96,97,98,99,100].

### 5.3. Anticancer Effect

In the context of melanoma treatment and prevention, natural plant compounds from the *Rosaceae* family, such as polyphenols, have been identified as having anticarcinogenic potential, including activities such as inducing apoptosis (programmed cell death), inhibiting cell proliferation, and reducing metastasis. These compounds may influence cell signaling, such as tumor proteins and DNA modifications [101]. Thus, significant effects on melanoma cell viability have been observed by testing phenolic extracts from *Malus domestica* apples on B16-F10 (murine melanoma) and SK-Mel-103 (human melanoma). Certain fractions reduced the viability of human melanoma cells by up to 38%, and in murine cells, antiproliferative effects were evident even at low extract concentrations. Phenolic compounds from apples may act through different mechanisms, including the induction of apoptosis, decreased COX-2 expression, and activation of caspases (caspase-3 and caspase-9), which are involved in the destruction of cancer cells. In addition, apple extracts demonstrated a significant protective effect on human fibroblast DNA against UV-induced DNA damage, suggesting a potential for the prevention of sun-induced skin cancer [102].

Euscaphic acid, one of the most abundant triterpene acids present in the soluble ethyl acetate fraction of a methanolic extract made from the leaves of the loquat, *Eriobotrya japonica*, was subjected to a two-step carcinogenesis assay on the skin of mice using DMBA (7,12-dimethylbenz[a]anthracene) as the initiator and TPA (12-O-tetradecanoylphorbol-13-acetate) as the promoter. Treatment with euscaphic acid reduced the incidence and number of papillomas compared to the control group, thus demonstrating that this compound has an inhibitory effect on tumor promotion [77]. Therefore, these triterpenes may act beneficially by preventing skin cancer, an effect demonstrated by the inhibition of tumor promotion in the mouse skin carcinogenesis model. In addition, megastigmane glycosides and procyanidins were investigated as potential antitumor agents of the same plant, *Eriobotrya japonica*. The anticancer effects are evidenced by inhibition of Epstein–Barr virus early antigen (EBV-EA) activation, a test for the identification of anticancer agents. Most notably, procyanidin B-2 and roseoside (a megastigmane glycoside) showed anti-tumor-promoting and anti-tumor-initiating effects in carcinogenesis experiments on mouse skin. These data may demonstrate the efficacy of natural compounds extracted from plants as promising sources of agents for cancer chemoprotection [103].

Loquat flowers (*Eriobotrya japonica* (Thunb.) Lindl.) are also a source of bioactive compounds. Following the analysis of the effect of an extract made from flowers, Chen Q. and coworkers highlighted the inhibitory effect on melanin synthesis in a concentration-dependent manner. The experimental results showed that the extract has a significant inhibitory effect on tyrosinase activity in mouse melanoma cells at concentrations of 50 and 100 μg/mL without inducing cytotoxicity, thus demonstrating the enzyme activity in a non-toxic manner. This effect was achieved by altering the structure and conformation of the enzyme without involving chelation of copper ions from its active center, suggesting an anticompetitive inhibition mechanism. Moreover, the treatment resulted in a significant decrease in the expression of proteins involved in melanin synthesis, such as TYR (tyrosinase), TRP1(tyrosinase-related protein 1), and TRP2 (tyrosinase-related protein 2), which contributed to the reduction in melanin formation in B16 cells. The significant ability of *Eriobotrya japonica* flower extract to reduce melanin synthesis may be a promising candidate for the development of cosmetic or pharmaceutical products against hyperpigmentation or the prevention of melanoma formation [104].

Hawthorn (*Crataegus*) is a promising agent for skin depigmentation treatments and melanoma treatment due to its antitumor, antioxidant, and melanogenesis inhibitory activities [105]. The total oligomer flavonoid (TOF) extract of *Crataegus azarolus* and its active compound (−)-epicatechin (EC) showed significant antiproliferative activity against B16F10 melanoma cells without affecting normal cells. These effects are attributed in part to the presence of flavonoids and polyphenols, and (−)-EC was identified as a compound with significant potential in inhibiting melanoma growth. TOF extract from *C. azarolus* demonstrated antitumor effects both in vitro (by inhibiting the growth of B16F10 melanoma cells) and in vivo (by significantly reducing tumor volume and weight in mouse models). These effects can be attributed to the presence of active compounds such as (−)-epicatechin, oleanolic acid, ursolic acid, and rutin. In addition, the TOF extract also showed significant antioxidant potential, protecting cells from oxidative damage caused by reactive oxygen species, suggesting that hawthorn (*C. azarolus*) could be a promising source of phytotherapeutic solutions for anticancer treatments and for the prevention of oxidative stress-mediated skin diseases [106].

Bioactive compounds such as C-glucosidic tannins (alienanin B and stenophyllanin A) and flavonoid glycosides (16 and 17) from the plants *Cowania mexicana* and *Coleogyne ramosissima* showed promising antitumor activity in in vivo mouse skin studies, significantly reducing the incidence and number of papillomas. Litospermoside, a nitrile glycoside, also showed complete inhibition of EBV-EA activation [107]. The Epstein–Barr virus, although a commonly known pathogen for tumors of the lymphatic system, also plays a significant role in skin oncogenesis, where it can cause various pathological processes [108]. According to the study conducted by Ito et al., these plants can be considered a valuable source of chemoprotective agents against skin cancer, having the potential to prevent the early stages of carcinogenesis induced by substances such as TPA, a well-known tumor promoter [107].

Another promising example is rose varieties (*Rosa* spp.), which are valuable sources of anthocyanins, flavonoids, and polyphenols with antioxidant and antiproliferative potential. Detailed chemical analyses by techniques such as HPLC-ESI-MS identified several types of anthocyanins that may contribute to their biological activity. In vitro studies demonstrated significant antiproliferative effects on the A375 cell line, indicating their anticarcinogenic potential. Increased concentrations of anthocyanins were correlated with an inhibition of tumor cell proliferation and a stimulatory effect on normal cells, suggesting a selective activity, which could be beneficial for treating cancer while protecting healthy cells [41].

In light of the above, different species and compounds of species from the *Rosaceae* family (e.g., *Eriobotrya japonica*, *Malus domestica*, *Rosa* spp.) show evidence of anticancer potential through antiproliferative and proapoptotic effects induced on cutaneous melanoma cells and observed in vivo [41,77,102].

### 5.4. Anti-Acne Effect

Oxidative stress plays a significant role in the development of acne and skin damage, and plant antioxidant compounds may help reduce this stress and thus improve acne [109]. Previous studies have shown that skin bacteria, such as *Cutibacterium acnes*, contribute to inflammation and the production of proinflammatory cytokines [110], and herbs such as *Cotoneaster*, which contain phenolic compounds with antioxidant properties, may help to combat these. In this context, the study conducted by Krzemińska B. et al. investigated the therapeutic potential of the species *Cotoneaster hsingshangensis* and *Cotoneaster hissaricus* in the treatment of dermatologic disorders, such as acne, by analyzing their biochemical composition and biological activities. LC-MS analysis identified 47 compounds, including flavonoids, phenolic acids, coumarins, cyanogenic glycosides, sphingolipids, and carbohydrates, supporting the hypothesis that *Cotoneaster* extracts have significant therapeutic potential due to their phytochemical composition. Quercetin derivatives, such as rutin, isoquercitrin, and hyperoside, were the most abundant, suggesting a significant antioxidant potential for these plants. These flavonoids are particularly important as they are known for their beneficial effects on skin health, including in acne treatments. Another significant compound identified was chlorogenic acid, with notable concentrations in both species, supporting their potential in the management of dermatological conditions [89].

Two other species of *Cotoneaster*—*Cotoneaster roseus* and *Cotoneaster nebrodensis*—were evaluated in the context of acne, intervening against oxidative stress by impairing antioxidant enzymes and increasing free radical levels. Both plants were shown to contain important active compounds, such as chlorogenic acid, isoquercitrin, (+)-catechin, and cinnamic acid, which are responsible for their beneficial biological activities, including antioxidant, anti-inflammatory, and antimicrobial activities. Following the DPPH assay, a dose-dependent antioxidant activity was observed, which could protect cells from oxidative damage, preventing inflammation and acne lesions. Of the two species, *C. nebrodensis* (especially the extract obtained from the fruit) showed the best performance results, having antioxidant and anti-inflammatory activity and an expected therapeutic index in terms of antibacterial and cytotoxic activity [91].

According to the studies described, *Cotoneaster* species, due to their high content of antioxidant and anti-inflammatory compounds (flavonoids, phenolic acids, coumarins, and catechins), may represent a potentially valuable source of natural agents in the prevention and treatment of acne [89,90,91].

### 5.5. Antityrosinase Effect

Tyrosinase is an enzyme found in a wide range of organisms, including animals, plants, and microorganisms. It plays a crucial role in melanin biosynthesis by catalyzing the first two steps: the hydroxylation of tyrosine to DOPA and the oxidation of DOPA to dopaquinone. At the stage of dopaquinone formation, the pathways for eumelanin and pheomelanin diverge. In the presence of thiol compounds like cysteine and glutathione, these molecules bind to dopaquinone, directing the biosynthesis toward pheomelanin. When L-tyrosine levels are low and cysteine levels are high, cysteine attaches to dopaquinone, forming cysteinyl dopa isomers [111].

In recent years, antityrosinase agents have garnered significant attention from researchers seeking substances that can both lighten the skin and address pigmentation disorders. Current studies show that various plant extracts and plant-derived compounds are potent tyrosinase inhibitors, effectively reducing excess melanin production in the epidermis. Notably, these compounds inhibit melanogenesis without causing cytotoxic or mutagenic effects on melanocytes, making them promising candidates for safe skin treatments [112].

Ursan triterpenoids isolated from the leaves of *Rubus fraxinifolius* were used to perform assays of elastase and tyrosinase inhibitory activities. The compounds determined were 2,3-O-ethylene glycol, 19-hydroxyur-12-en-23,28-dioic acid and 2,3-O-propanediol, 19-hydroxyur-12-en-28-oic acid, to be characterized by spectroscopic analysis. These inhibited elastase with IC50 122.199 and 98.22 pg/mL and also inhibited tyrosinase with IC50 207.79 and 221.51 pg/mL, respectively. Both compounds had lower inhibitory activity than the positive control, oleanolic acid, which had an IC50 value of 90.39 pg/mL [113].

Furthermore, total oligomeric flavonoid (TOF) extract from *Crataegus azarolus* and epicatechin, an active compound of the extract, may influence melanogenesis by inhibiting tyrosinase activity, thus having potential applicability in skin depigmentation and melanoma treatments. Epicatechin, due to its chemical structure, can act as a competitive inhibitor of tyrosinase, preventing the binding of the natural substrate (L-DOPA) to the active site of the enzyme, which contributes to decreased melanin production [106].

*Rubus chingii* Hu (Chinese raspberry) is also part of the *Rosaceae* family and is considered an important plant in the context of cosmetics due to its tyrosinase inhibitory properties, making it a potential ingredient for skin-lightening and antiaging products [114]. One of the active compounds in this plant is tyliroside, an acylated flavonoid that has demonstrated multiple benefits for the skin due to its ability to inhibit tyrosinase activity and reduce melanin production in a dose-dependent manner, with a greater impact than arbutin, a commonly used ingredient in the cosmetic industry for skin brightening. It has shown significant efficacy in reducing melaninogenesis without exhibiting significant cytotoxic effects on B16 cells, even at low concentrations [115].

An article by Nile S.H. et al. demonstrated that fractions from applesauce (AP) and triterpene acids (TAAs) exhibit potent tyrosinase inhibitory activities, making them potential candidates for hyperpigmentation and melanoma-related treatments. Among the AP solvent fractions, the methanol extract exhibited the most potent tyrosinase inhibition, with an IC50 value of 13.2 μg/mL. TAAs, particularly ursolic acid and betulinic acid, were found to be more effective inhibitors than the commonly used kojic acid, with IC50 values of 8.4 μg/mL and 10.1 μg/mL, respectively. Kinetic studies of ursolic acid showed competitive inhibition, meaning that it competes with the substrate for binding to the active site of the tyrosinase enzyme [116].

Tyrosinase, a key enzyme involved in melanin production, is being studied for its activity and also to find possible inhibitors of its activity. The identification of active constituents in Prunus species flowers and the preliminary investigation of the signaling pathway of tyrosinase expression are the objects of the study. Of the two species, *P. persica* (PP) and *P. yedoensis* (PY), *P. persica* extract demonstrated the most potent inhibitory effect on tyrosinase, with 46.0% and 52.1% inhibition at 200 and 500 μg/mL. In contrast, *P. yedoensis* was found to be the most effective in reducing melanin content in B16 cells, with significant suppression of melanin production at concentrations of 100 and 500 μg/mL without cytotoxicity. It is hypothesized that afzelin and naringenin, isolated from *P. persica*, is responsible for the tyrosinase inhibitory and melanogenesis-suppressing effects through the inhibition of the p38 MAPK pathway. Both compounds suppress the expression of Mitf, the key transcription factor for tyrosinase biosynthesis, and reduce the phosphorylation of p38 MAPK, unlike the α-MSH pathway, which typically promotes melanogenesis. Additionally, both compounds were found to slightly suppress Akt, a protein known to promote protein biosynthesis, further supporting their inhibitory effects on melanogenesis [117].

*Rosa rugosa* Thunb. var. *plena regal* is a variety of rose that contains several active compounds, such as essential oils, polyphenols, flavonoids, and anthocyanins, which are recognized for their multiple biological benefits, including antimicrobial, anti-inflammatory, and antioxidant activities. RFCS (rose flower cell sap), a by-product of rose flowers, is being studied for its tyrosinase inhibitory effect due to its high flavonoid content. RFCS exhibits significant tyrosinase inhibitory activities, with an IC50 of 570 μg/mL, and the flavonoid compounds in RFCS are more effective than kojic acid in inhibiting this enzyme, contributing to its therapeutic potential [118].

Tyrosinase inhibitory plant extracts and compounds, such as flavonoids, triterpenoids, and phenolic acids, act either by competitive inhibition at the active center of the enzyme or by modulating signaling pathways involved in MITF gene expression (such as p38 MAPK), showing promising therapeutic potential for the treatment of cutaneous hyperpigmentation and other melanogenetic disorders [113,114,115,116,117,118].

### 5.6. Natural Products Protect the Molecules of the Extracellular Matrix (Elastin, Collagen, and Hyaluronic Acid)

Ceramides in the human stratum corneum can be categorized into 11 groups based on the structure of fatty acids and sphingoid bases. These ceramides play an essential role in the skin’s barrier function. They are synthesized via two main pathways, the glucosylceramide pathway and the sphingomyelin pathway, each involving specific enzymes [119]. In this context, strawberry seed extract (SSE) and tilirozides were studied for their impact on the expression of enzymes involved in ceramide synthesis, and the results suggest that the extract significantly stimulates ceramide synthesis, especially of those in the glucosylceramide pathway, by increasing the expression of GCS (Glucosylceramide Synthase) and GBA (Glucosylceramidase Beta) enzymes, whereas tilirozide has a weaker and more restricted effect on this process. The PPARα, β/δ, and γ activators are involved in stimulating the synthesis of epidermal lipids, including ceramides, and accelerating the recovery of the skin permeability barrier. These findings suggest that SSE could be effective in improving skin barrier function and in treatments for ceramide-deficient skin conditions such as atopic dermatitis and xerosis [120]. *Eriobotrya japonica* (loquat), a plant with various medicinal properties, also demonstrates the bioactive potential of triterpenoids extracted from the leaves. In particular, corosolic acid and pomolic acid significantly stimulate the production of hyaluronic acid in human dermal fibroblasts, which may help to maintain skin moisturization, increase elasticity, and aid in the wound-healing process. These compounds could also inhibit hyaluronidase, an enzyme that degrades hyaluronic acid, thereby helping to protect and maintain skin health [10]. Cherry blossom flowers (*Prunus lannesiana*) could have a potential effect against skin aging by protecting essential skin structures from damage caused by glycation and the negative effects of AGEs [121]. The accumulation of AGEs (advanced glycation end products) in the body is associated with several conditions, as glycation can damage protein structures in the body, such as collagen and elastin in the skin, leading to loss of elasticity and premature aging of the skin [122]. Cherry blossom flowers contain bioactive compounds, such as cinnamoyl glucoside derivatives and flavonoid glucosides, which have a significant effect on preventing the formation of AGEs and protecting fibroblasts from AGE-induced apoptosis (cell death). The main components of cherry blossom flowers, in particular 1-O-(E)-caffeoyl-β-D-glucopyranoside, have demonstrated the ability to inhibit AGEs production in fibroblasts and to recover glycation-induced collagen network formation [121].

*Rubus idaeus* lipid-soluble extract (RCLE) has a significant effect in promoting skin hydration and protecting epidermal cells against excessive water loss. It stimulates the expression of important genes involved in water balance and skin hydration. In a study by Tito et al. on human keratinocytes (HaCaT), treatment with RCLE resulted in increased expression of the AQP3 (aquaporin 3), FLG (filaggrin), and INV (involucrin) genes, which are essential for maintaining proper water balance in the skin. It was observed to stimulate the production of collagen and fibronectin, proteins essential for maintaining skin structure and elasticity. The extract also helps in the formation of elastin fibers and the synthesis of proteins that ensure skin cohesiveness and resilience, and it is effective in combating signs of skin aging by maintaining an optimal water balance and strengthening the extracellular matrix structure [123].

Thus, bioactive compounds from plants such as *Rubus idaeus*, *Prunus lannesiana*, *Eriobotrya japonica,* and strawberry seed extract may help improve skin barrier function, hydration, and protection against aging by stimulating the expression of key genes involved in the synthesis of ceramides, hyaluronic acid, and structural proteins and by inhibiting advanced glycation processes [120,121,122,123].

### 5.7. Antioxidant Effect

Apple pomace (AP), a waste product generated in the industrial processing of apples, is a valuable source of bioactive compounds (such as polyphenols and pentacyclic triterpenes) that can be recovered and used to create value-added products. These substances have various health benefits, including antioxidant, antimicrobial, and anti-inflammatory properties, and are suitable for use in functional foods, cosmetics, and pharmaceuticals. Their efficient processing can help both to reduce the environmental impact of waste and to develop products with clinical and pharmacological benefits. Phenolic compounds, which are secondary plant metabolites, possess antioxidant properties and play an important role in free radical stabilization. Extraction methods and choice of solvents to maximize the recovery of valuable phenolic compounds from plant by-products, such as PA, is essential in determining the content of these compounds. Methanol shows the highest total phenolic content (TPC), while hexane has the lowest content. The variation in phenolic composition can also be influenced by factors such as apple variety, genotype, and environmental conditions [116].

Also, a study by Fattouch et al. focused on the properties of extracts from an important genus, genus Malus, of the subfamily *Pomoideae*. The fruit extracts were obtained by a mixed solvent water/acetone (3:1) extraction method to reveal the main active compounds. It was thus shown that quince, apple, and pear fruits can be excellent sources of polyphenols with antioxidant properties, especially in their peel. Their high content of compounds such as chlorogenic acid, rutin, kaempferol, and phloridzin makes them effective in fighting free radicals and protecting the body from oxidative stress. Apples appear to be particularly valuable due to their higher anthocyanin and flavonoid content, while quinces and pears are also important sources of antioxidants, especially in the peel [124].

According to experimental data, the extract of *Malus pumila* cv. *Annurca* has the potential to combat hair loss through its antioxidant activity and by stimulating hair growth. The study investigated the beneficial effects of this extract, in particular by reducing reactive oxygen species (ROS) and stimulating the expression of factors that regulate hair growth. Annurca apple, known for its high content of procyanidin B2, an active compound that promotes hair growth, may be a promising solution in treatments to prevent and treat hair loss. Chlorogenic acid, another phenolic compound with significant effects in preventing hair loss, was also identified in the extract. In addition, the extract has potent antioxidant activity, acting on the reduction of oxidative stress by increasing the activity of the antioxidant enzymes SOD and CAT, inducing the expression of β-catenin, and increasing the expression of follicular growth factors such as FGF-2, which play a crucial role in the development and regeneration of hair follicles [125].

The use of anthocyanins and flavonoids with antioxidant and sunscreen activities in cosmetic formulations to protect the skin against UV rays and free radicals is increasingly common [126]. In vitro antioxidant activity of raspberry and blackberry extracts was evaluated using a DPPH assay. The results showed that raspberry extract inhibited free radicals by 82.33% and blackberry extract by 74.01% compared to quercetin, an effective antioxidant flavonoid used as a positive control. Although the antioxidant activity of the extracts was lower than that of quercetin, the presence of flavonoids in these extracts was responsible for their antioxidant activity [127].

Dermal fibroblasts are key cells in maintaining skin structure and function, and oxidative stress can induce significant damage to these cells, including apoptosis (programmed cell death), which contributes to skin aging and loss of skin elasticity [128]. In this context, strawberry extract, rich in anthocyanins and other phenolic compounds, may protect fibroblasts from oxidative stress and prevent apoptosis induced by ROS. Strawberry anthocyanins, through their antioxidant properties, reduce ROS levels in cells and thus protect fibroblasts from oxidative damage. They may prevent the activation of apoptosis signaling pathways and protect cells from premature cell death, thus helping to maintain fibroblast health and function. In addition, strawberry extract may help protect cell membranes and mitochondrial functionality, which are essential in preventing apoptosis caused by oxidative stress [129].

The extract made from *Pourthiaea villosa* was analyzed by UHPLC-LTQ-IT-IT-MS/MS and multivariate statistical analysis, and biologically active secondary compounds such as phenolics, polyols, and flavonoids were determined. *Pourthiaea villosa* (Thunb.) Decne. (PVDE) extract demonstrated significant protective effects against H_2_O_2_-induced oxidative stress in human dermal fibroblasts (HDFs) without inducing cytotoxicity. Treatment with PVDE reduced ROS production in a dose-dependent manner and increased the levels of certain antioxidant enzymes, such as SOD1 and SOD2, suggesting that PVDE protects cells by activating the antioxidant enzyme system. These results indicate a protective potential of PVDE against oxidative stress [99].

Extracts of *Rosa damascena* obtained by different extraction methods (aqueous ethanol or ethyl acetate) have shown a strong antioxidant effect, which is due to the presence of phenolic compounds. Major compounds, such as flavonoids (kaempferol and quercetin) and phenolic acids, are key factors in neutralizing free radicals and protecting the skin against oxidative stress. DPPH, ABTS, and FRAP radical scavenging assays showed significant antioxidant activity of the extracts, and the phenolic-enriched fraction had a higher radical scavenging capacity compared to the original extract, suggesting a potentiation of the antioxidant effect by re-extraction [130]. This antioxidant effect is beneficial for skin protection, as oxidative stress plays a major role in premature aging of the skin and the development of dermatologic disorders [131].

Below is a summary table (Table 2) showing the species of the *Rosaceae* family and the main compounds responsible for their antioxidant activity.

### 5.8. UV-Protecting Effects

Exposure to ultraviolet (UV) radiation from the sun causes considerable damage to the skin, contributing to premature aging, hyperpigmentation, and oxidative stress that affects the skin’s natural balance. UV radiation stimulates the production of reactive oxygen species, which accelerates skin damage. Although sunscreens remain the main defense against UV rays, formulations containing chemicals can have side effects, such as allergic reactions. To remedy these side effects, the use of natural ingredients in sunscreens has gained increasing attention [132].

The study by Her Y. and coworkers thereby demonstrated the effects of *Aronia melanocarpa* extract (AME) on skin damage induced by UVB radiation exposure in terms of collagen changes and MMPs activity, which are enzymes involved in extracellular matrix (ECM) degradation. UVB irradiation can lead to significant damage to the skin, including destruction of dermal fibroblasts, loss of collagen structure, and increased levels of MMPs, which contribute to collagen degradation. These processes are linked to the production of ROS, which trigger inflammation and damage the skin’s supporting structures. In the study, the authors topically applied AME to the dorsal skin of mice exposed to UVB radiation and observed that the extract attenuated typical UVB-induced changes such as epidermal thickening, fibroblast loss, and collagen disruption. Furthermore, the application of AME resulted in a significant decrease in the activity of MMPs, particularly MMP-1 and MMP-3, which are involved in collagen degradation. This protective effect was supported by an increase in collagen production in AME-treated skin compared to the vehicle-only group. Another important aspect of the study was the identification of phenolic compounds in AME, including chlorogenic acid and rutin, which were detected by high-performance liquid chromatography (HPLC). These compounds are known for their antioxidant and anti-inflammatory properties, and previous research has shown that chlorogenic acid protects against UVB-induced damage by reducing ROS and preventing DNA damage. Rutin has also shown protective effects on fibroblasts, inhibiting inflammatory responses and reducing UVB-induced damage by inhibiting inflammatory enzymes such as COX-2 and iNOS. The study thus suggests that *Aronia melanocarpa* extract has a significant photoprotective effect, protecting the skin from UVB damage by inhibiting collagen degradation and reducing MMP activity, making it a promising candidate for the development of UV-protective cosmetic or pharmaceutical products [133].

Another study explored whether ethanolic extracts from *Rosa multiflora* flowers (RMF) and the fractions obtained have a photoprotective effect on UV-aged skin. Extracts rich in polyphenols and flavonoids may prevent UV-induced skin damage in human and mouse skin cells due to their antioxidant and anti-inflammatory properties. Polyphenols can suppress the production of ROS and proinflammatory cytokines (such as IL-6 and IL-8), which are generally upregulated after UV irradiation. Also, UV-induced oxidative stress can lead to collagen degeneration, which contributes to wrinkle formation and skin aging. RMF extracts also help to balance collagen degradation and synthesis by inhibiting matrix metalloproteinase, blocking key signaling pathways NF-kB and MAPK (ERK, JNK, p38) expression, and promoting procollagen production, thereby preserving the extracellular matrix (ECM) of the skin and preventing photoaging. In addition, UV exposure of mice induced epidermal thickening, inflammation, and MMP expression, but these effects were significantly reduced when mice were treated with a mixture of REA and RBT. The study found several phenolic compounds in RMF extracts, including quercitrin, a flavonoid with known antioxidant, anti-inflammatory, and antiproliferative properties. Quercitrin was found to reduce MMP-1 levels and increase procollagen type I levels in UV-irradiated human dermal fibroblasts (HDFs), suggesting that it plays a role in the photoprotective effects of RMF extracts [134].

Exposure to UVB radiation can cause significant skin damage, including skin cancer and photoaging. This damage is caused by the formation of DNA damage (CPD), lipid peroxidation, and activation of MMPs, which degrade the extracellular matrix. Malus sp. extract and rutin, an active compound in the extract, have shown protective effects against UVB-induced damage in radiation-exposed skin by preventing caspase-3 activation and the formation of CPDs (pyrimidine dimers), which are signs of DNA damage. Both *Malus* sp. extract and rutin significantly inhibited CPD formation and cell apoptosis and were associated with antioxidant and reactive oxygen species (ROS) scavenging activities, thus protecting the skin from the negative effects of UVB radiation. Malus sp. extract and rutin, included in topical formulations, demonstrated photochemopreventive effects by reducing lipid peroxidation and MMP activity induced by UVB radiation. These effects suggest that these compounds help to balance the oxidative stress of the skin, protecting it against structural damage and aging processes, by reducing lipid peroxidation and inhibiting the activity of MMPs involved in extracellular matrix degradation [135].

UV irradiation can induce various skin lesions due to excessive melanin synthesis [136]. A study by Tan H. et al. investigated the potential involvement of triterpenoids present in *Eriobotrya japonica* leaves in skin-related bioactivity. The potential antimelanogenesis effect of 18 triterpenoids from a methanolic extract of *Eriobotrya japonica* leaves was tested and found to exhibit inhibitory effects on melanin synthesis. Of the tested compounds, those with high selectivity index, such as ursolic acid and maslinic acid, are promising for use in the prevention and treatment of UV-induced skin lesions such as age spots and melasma [10].

Plant extracts rich in polyphenols, flavonoids, and triterpenoids, such as those from *Aronia melanocarpa*, *Rosa multiflora*, *Malus* sp., and *Eriobotrya japonica,* provide effective protection against photoaging by reducing oxidative stress, inhibiting degradative enzymes, and preventing UV-induced DNA damage, supporting the development of natural photoprotective cosmetic formulations [10,132,133,134,135,136].

### 5.9. Antiaging Effect

The functional properties of the skin depend on the quality and condition of the collagen present in the dermis, which is why collagen secreted by normal human dermal fibroblasts (NHDFs) plays important roles in cell–cell adhesion, cell proliferation, and cell differentiation [137]. The results of some studies indicate that triterpenoids such as methyl corosolate and corosolic acid showed a particularly potent stimulation of dermal collagen production from fibroblasts [10].

The number of fibroblasts, the most common connective tissue cells, decreases with age, and this is thought to be due to reduced proliferation. In this respect, the natural substances present in the rich extract of callus (A2 line) of *Chaenomeles japonica* focus on the beneficial effects on the biological activities of skin fibroblasts. The positive effect on fibroblasts may be related to the compounds present in the biomass, in which pentacyclic triterpenoids (ursolic acid, oleanolic acid) and phenolic compounds (chlorogenic acid) predominate. Ursolic acid stimulates collagen production in dermal fibroblasts, contributing to antiaging effects, and chlorogenic acid has strong antioxidant activity, protecting fibroblasts from oxidative stress. The extract promotes the growth and proliferation of human skin fibroblasts, supporting skin regeneration [138].

*Cydonia oblonga* (quince) stands out as a valuable natural ingredient in antiaging cosmetics due to its multiple beneficial skincare properties. Extracts of quince peel and pulp have shown significant potential to inhibit tyrosinase, a crucial enzyme in the melanogenesis process, thereby helping to reduce hyperpigmentation and improve the even appearance of the skin. These effects are supported by the presence of flavonoids and anthocyanins, compounds with a powerful antioxidant effect that protect the skin from oxidative damage and reduce the visible signs of aging [139]. In addition, recent studies have highlighted the ability of quince extract to improve skin elasticity and increase moisture content, which are essential for maintaining youthful, moisturized skin. Thus, *Cydonia oblonga* can be considered an effective solution to combat the signs of aging, having a positive impact on both the aesthetic appearance and long-term health of the skin [140]. Overall, active compounds from plants such as *Chaenomeles japonica*, *Cydonia oblonga*, and triterpenoids from *Eriobotrya japonica* stimulate collagen production, protect fibroblasts, and help maintain skin elasticity and regeneration, highlighting their potential in preventing and combating signs of skin aging [138,139,140].

### 5.10. Antipsoriasis Effect

Psoriasis is an inflammatory, chronic skin disease. Worldwide, the prevalence of psoriasis ranges between 2 and 3%, with an interindividual variation depending on multiple factors such as age, gender, ethnicity, and genetic profile [141]. Clinically, it is distinguished by the appearance of itchy erythematous plaque, which could have a various anatomical distribution; usually, the most targeted areas are the elbows, trunk, scalp, and sacral area [142]. The etiopathogenesis of psoriasis is not yet completely elucidated, but it is well-known that genetic, immunologic, and inflammatory disorders play an essential role in the occurrence of this condition [143]. Prior clinical studies on patients with psoriasis have shown an elevated level of cytokines like interleukins (IL-17, IL-36, IL-23, IL-22), monocytes, and neutrophils. However, T-cell lymphocyte dysfunction, especially Th-17, is considered the main cause of this disease [144]. These cytokines could act in keratinocytes in association with TNF-α; consequently, the cells’ proliferation is initiated [145]. A study conducted by Xuyan et al. on an animal model revealed that the bioactive phytocompounds from the leaves of *Eriobotrya japonica*, particularly the pentacyclic triterpenes methyl corosalate, uvaol, and oleanolic acid, have a favorable effect on psoriasis progression and severity. These pentacyclic triterpenes seem to have a marked suppressive action on the expression of Retinoid-related Orphan Receptor γt (ROR γt) mRNA, and in this manner, they inhibit Th17 activity and the keratinocyte release of psoriasis-related proteins, consequently inhibiting the hyperproliferation of keratinocytes and reducing inflammation [146].

Another species that presents potential in the therapy of psoriasis is *Rosa damascena* Mill. (Damask rose). Through its phytocompounds, such as flavonoids, phenolic acids, and essential oils, *Rosa damascena* exerts therapeutic effects. Quercetin, a bioactive compound from this species, can suppress NF-κB activity and reduce oxidative stress, while kaempferol, from *Rosa damascena*, can downregulate the expression of COX-2 and inducible Nitric Oxide Synthase (iNOS), which can impact T-cell proliferation and cytokine production. Gallic acid, another active phytocompound in *Rosa damascena* extracts, exhibits anti-inflammatory effects by reducing the production of proinflammatory cytokines. Meanwhile, anthocyanins promote skin barrier function and scavenge free radicals, offering a protective effect against external factors that may exacerbate psoriasis symptoms. The application of *R*. *damascena* creams in vivo resulted in a significant reduction in psoriasis symptoms, supporting the use of *Rosa damascena* as a promising option for the development of effective natural topical therapies for the management of psoriasis [18].

In analyzing the studies described, which show significant anti-inflammatory and immunomodulatory effects, natural topical therapies may be promising in relieving the symptoms of psoriasis, an increasingly common disorder, due to bioactive compounds such as triterpenoids, flavonoids, and phenolic acids [142,143,144,145,146].

### 5.11. Pharmacological Insights into the Skin-Related Effects of Rosaceae Family Species

In view of the enormous findings underpinning the therapeutic potential of the species and compounds found in the *Rosaceae* family in the field of skin diseases, a list of the existing evidence for biological activities in this regard can be found in Table 3.

## 6. Modern Pharmaceutical Formulations Using *Rosaceae* Extracts with Dermatological Potential

The skin, being in direct contact with many cosmetic and medical–aesthetic products containing nanoparticles, is a critical route of exposure, but the precise mechanisms of the interaction between nanoparticles and skin structures are still poorly elucidated. It is essential to assess long-term skin effects, especially in the context of biostimulants and nanomaterials used in aesthetic medicine [167]. A recent study by Wang et al. demonstrated that gold nanoparticles synthesized from *Rubus rosifolius* (RR-AuNPs) exhibit remarkable anti-inflammatory activity on human keratinocytes without inducing cytotoxicity. RR-AuNPs significantly reduced the secretion of proinflammatory cytokines and chemokines, as well as oxidative stress induced by TNF-α and IFN-γ. These effects were associated with the inhibition of MAPK and NF-κB signaling pathways, highlighting their potential as topical anti-inflammatory agents in nanotechnology-based dermatological or aesthetic treatments [168]. Another recent study by Cima et al. demonstrated the efficacy of dermato-cosmetic hydrogels formulated with ethanolic macerates of *Rosa canina* (fruits and flowers), which show remarkable antioxidant activity, supported by a high content of polyphenolic and flavonoid compounds. The formulation, obtained with 60% ethanolic macerate of rosehip fruit, showed the best results in DPPH and FRAP tests, highlighting its ability to combat oxidative stress associated with skin aging, erythema, and photoaging. The smooth texture, pseudoplastic rheological behavior, and physicochemical stability recommend this type of hydrogel as a promising vehicle for bioactive compounds in modern dermato-cosmetic and cosmeceutical products [169]. Moreover, *Rosa canina* seed oil proved to be a valuable source of bioactive compounds with regenerative potential, and its formulation in stable phospholipid liposomes, by the proliposome technique, allowed the protection of sensitive components and the maintenance of physicochemical characteristics essential for topical application. Although the antioxidant activity of these liposomes was moderate and no antimicrobial effects were observed, their elevated stability and the beneficial interaction between phospholipids and seed oil support their use in dermato-cosmetic formulations for skin rejuvenation, especially in the context of damaged or aged skincare [170]. Silver nanoparticles (AgNPs) have been extensively investigated for their antibacterial, anti-inflammatory, and immunomodulatory activity. In a recent study presented by Majid S. Jabir and colleagues, AgNPs biosynthesized using *Eriobotrya japonica* leaf extract demonstrated a significant inflammation-suppressing effect both in vitro and in vivo. Treatment with AgNPs significantly reduced the levels of IL-1β and IL-6, key proinflammatory cytokines involved in inflammatory skin pathology, and in animal models, AgNPs reduced systemic inflammation, including immunological parameters associated with allergic reactions. The proposed mechanisms involve inhibition of MAPK and NF-κB signaling pathways, both of which are major activators of tissue inflammation [171]. These findings support the potential use of AgNPs in dermato-cosmetic formulations, particularly in the context of inflammatory skin conditions such as acne, where chronic inflammation and bacterial colonization are essential elements of pathogenesis [172]. 

Given the great therapeutic potential of biosynthesized nanoparticles and plant extracts formulated in modern delivery systems, it is fundamental that future research should focus on long-term in vivo studies, dermatological safety assessment, and local bioavailability of these compounds. In addition, the incorporation of these biocomponents into personalized dermato-cosmetic products could offer new perspectives in non-invasive aesthetic medicine, with an increased focus on the prevention of skin aging and the management of chronic inflammatory conditions.

## 7. Conclusions

The present review showed the significant potential of plants of the *Rosaceae* family (e.g., *Eriobotrya japonica*, *Rosa damascena)* in the development of cosmetics and pharmaceuticals for skin protection and care. The active substances in these plants, such as chlorogenic acid, rutin, quercitrin, and triterpenoids, have demonstrated antioxidant and anti-inflammatory effects, protecting the skin from UV-induced damage, reducing oxidative stress, and inhibiting inflammatory processes. Studies suggest that extracts of *Aronia melanocarpa* and *Rosa multiflora* may prevent collagen degradation and reduce MMP activity, thereby protecting the skin’s extracellular matrix and preventing photoaging. Also, phenolic compounds in these extracts, such as quercitrin, play a key role in stimulating collagen production and reducing visible signs of skin aging. In addition, triterpenoids, such as ursolic acid and maslinic acid, present in *Eriobotrya japonica* extracts, have demonstrated a significant antimelanogenetic effect, indicating their potential in treating UV-induced skin lesions such as age spots and melasma. Concerning psoriasis, a frequent condition, *Rosa damascena* and *Rosa canina* have shown beneficial effects when used in topical formulations. In conclusion, the *Rosaceae* family represents a promising candidate for cutaneous organ disorders with a huge array of biological properties (antioxidant, anti-inflammatory, antiaging, anticancer, antityrosinase). Moreover, the integration of *Rosaceae* plant extracts into advanced dermato-cosmetic delivery systems, such as nanocarriers (e.g., gold or silver nanoparticles, liposomes, hydrogels), enhances their bioavailability, stability, and efficacy. Future research directions should focus on the elaboration of topical pharmaceutical formulations incorporating extracts of *Rosaceae* species, taking into account the results observed in preclinical studies.

## Figures and Tables

**Table 1:** Presentation of the main mechanisms of action on skin diseases produced by species of the *Rosaceae* family.

Species	Anti-Inflammatory Mechanisms of Action	Targeted Skin Conditions	References
*Eriobotrya japonica*	↓ NF-κB, ↓ p38 MAPK, ↓ ERK	Inflammation, edema, acne	[76]
*Filipendula palmata*	↓ Chemokine genes/proteins (via MAPK and NF-κB pathways)	Burns, skin inflammation	[79]
*Cydonia oblonga*	↓ NF-κB, ↓ p38 MAPK, ↑ AKT, ↓ COX-2	General inflammation, skin aging	[81,82]
*Pyrus ussuriensis*	↓ NO, ↓ IL-6, ↓ IL-1β	Atopic dermatitis	[84]
*Crataegus* spp.	↓ TNF-α, ↓ IL-1β, ↓ IL-6, ↓ IL-33, ↓ NO, ↓ PGE2, ↑ IL-10	Chronic inflammation, wound healing	[85,86]
*Rubus idaeus*	↓ COX, ↓ LOX	Inflammatory and oxidative skin damage	[87]
*Rosa damascena*	↓ Inflammation gene Expression	Wound healing, antiaging	[88]
*Cotoneaster* spp.	↓ Hyaluronidase, ↓ COX-1/2, ↓ LOX, ↓ prostaglandins	Acne, oxidative stress, skin inflammation	[89,91]
*Rosa multiflora*	Downregulation of iNOS and COX-2 expression	Atopic dermatitis	[93]

↓—inhibition/decrease in activity or expression, ↑—activation/increase in activity, NF-κB—Nuclear Factor Kappa B, MAPK—Mitogen-Activated Protein Kinase, ERK—Extracellular Signal-Regulated Kinase, COX—cyclooxygenase (COX-1, COX-2), LOX—lipoxygenase, AKT—Protein Kinase B, NO—nitric oxide, TNF-α-—tumor necrosis factor-alpha, IL-1β—Interleukin-1 beta, IL-6—Interleukin-6, IL—Interleukin-33, PGE2—prostaglandin E2, ↑ IL-10—Interleukin-10.

**Table 2:** Antioxidant activity of *Rosaceae* species.

Species	Main Active Compounds	Antioxidant Mechanism of Action	References
*Malus domestica (apple pomace)*	Polyphenols, triterpenes	↓ ROS, stabilizes free radicals	[116]
*Malus pumila* cv. *Annurca*	Procyanidin B2, chlorogenic acid	↑ SOD, ↑ CAT, ↑ FGF-2, ↓ ROS	[125]
*Rubus idaeus (raspberry)*	Flavonoids	Inhibits DPPH by 82.33%	[127]
*Rubus fruticosus (blackberry)*	Flavonoids	Inhibits DPPH by 74.01%	[127]
*Fragaria vesca (strawberry)*	Anthocyanins, polyphenols	Protects fibroblasts, ↓ROS, prevents apoptosis	[129]
*Pourthiaea villosa*	Flavonoids	↓ ROS, ↑ SOD1/SOD2, protection against oxidative stress	[99]
*Rosa damascene*	Kaempferol, quercetin, phenolic acids	Strong scavenging of DPPH, ABTS, FRAP	[130]

↓—inhibition/decrease in activity or expression, ↑—activation/increase in activity, ROS—reactive oxygen species, SOD—superoxide dismutase, CAT—catalase, FGF-2—Fibroblast Growth Factor 2, DPPH—2,2-diphenyl-1-picrylhydrazyl, ABTS—2,2′-azino-bis(3-ethylbenzothiazoline-6-sulfonic acid), FRAP—Ferric-Reducing Antioxidant Power.

**Table 3:** Studies involving the potential of *Rosaceae* family members in skin diseases.

Genus	Plant Species	Biological Effect	Results	References
*Rubus*	*Rubus parvifolius* L.	▪ Antimelanoma	For 24, 48, and 72 h, A375 cells were subjected to different concentrations of total saponin extract (ranging from 0.1 to 300 µg/mL). After 72 h, TSRP at 100 µg/mL caused a reduction in cell viability of approximately 60% when compared to the control group. The migration distance was significantly decreased compared to untreated cells after 48 h of treatment with extract. The establishment of xenograft tumors was performed by inoculating BALB/C nude mice with A375 cells. After the tumor developed, mice were given TSRP intraperitoneally in doses of 25, 50, and 100 mg/kg daily for a period of 14 days. The TSRP treatment had a dose-dependent effect on tumor volume and weight compared to the vehicle-treated group.	[147]
	*Rubus* *coreanus* L.	▪ Anti-photoaging	Ethanolic extract pretreatment with concentrations ranging from 1 to 10 µg/mL inhibited the production of MMP-1, MMP-8, and MMP-13 in a dose-dependent manner. The suppression of these enzymes, which are involved in collagen degradation, suggests that ethanolic extract has a protective effect on collagen breakdown. UV-B-exposed dermal fibroblasts saw a significant increase in type I procollagen and collagen levels after exposure to the extract. This demonstrates that extract not only prevents collagen degradation but also stimulates collagen synthesis, which contributes to the maintenance of the extracellular matrix integrity.	[148]
		▪ Antioxidant and anti-inflammatory activity	Significant antioxidant properties by reducing intracellular reactive oxygen species (ROS) levels and enhancing the activity of antioxidant enzymes such as catalase, superoxide dismutase, and glutathione peroxidase in tumor necrosis factor-α (TNF-α) and interferon-γ (IFN-γ)-stimulated HaCaT cells (human keratinocytes). Led to a decrease in the mRNA expression of proinflammatory cytokines, including TNF-α, interleukin-1β (IL-1β), and interleukin-6 (IL-6). Increased the mRNA expression levels of filaggrin and involucrin, proteins essential for skin barrier function, in TNF-α/IFN-γ-stimulated HaCaT cells.	[149]
		▪ Anti-inflammatory, antiallergic effects	Reduced the release of β-hexosaminidase and histamine from human mast cells (HMC-1) stimulated with compound 48/80. Led to a significant decrease in both protein and mRNA expression levels of various cytokines (IL-4, IL-5, IL-12, IFN-γ, TNF-α, and TARC) and IgE in the AD mouse model.	[150]
	*Rubus occidentalis* L.	▪ Antiaging, antimelanogenesis effects	In human dermal fibroblast cells (CCD-986sk), the extract significantly inhibited MMP-1 activity by 18% and increased type I procollagen synthesis by 25%. Significantly inhibited α-melanocyte-stimulating hormone-induced melanin synthesis and tyrosinase activity, indicating potential skin-whitening effects.	[151]
	*Rubus* *sanctus* S.	▪ Wound-healing activity	The methanol extract ointment (1% concentration) significantly increased wound tensile strength compared to control groups, indicating enhanced wound healing. Notable reduction in wound area and accelerated wound contraction, leading to faster re-epithelialization compared to controls.	[152]
	*Rubus* *idaeus* L.	▪ Antiaging activity	Increased the expression of matrix metalloproteinases (MMPs), enzymes that degrade collagen and contribute to skin aging. Treatment with red raspberry extract significantly reduced UVB-induced MMP-1 expression in NHDFs. Suppressed the activation of Mitogen-Activated Protein Kinases (MAPKs) and activator protein-1 (AP-1), as well as Nuclear Factor Kappa-light-chain-enhancer of activated B cells (NF-κB), pathways known to upregulate MMP expression.	[153]
	*Rubus* *caesius* L.	▪ Antioxidant, antihyaluronidase, antimicrobial activities	Both water and ethanol extracts from the stems inhibited hyaluronidase activity, with IC_50_ values of 55.24 ± 3.21 µg/mL and 68.7 ± 1.61 µg/mL, respectively. Both water and ethanol extracts demonstrated antimicrobial efficacy against *Clostridium bifermentans*, *Clostridium sporogenes*, and *Enterococcus faecalis*.	[154]
*Prunus*	*Prunus* *spinosa* L.	▪ Antioxidant, antimicrobial, anticancer activities	The hydro-ethanolic extract did not exhibit any significant hemolysis, which indicates compatibility with erythrocytes, and did not exhibit cytotoxic effects on fibroblasts (3T3), keratinocytes (HaCaT), and carcinoma cells (A431) at concentrations up to 200 µg/mL. Showed antibacterial activity against *S. aureus* and *S. epidermidis* (16 mg/mL).	[155]
*Potentilla*	*Potentilla anserina*	▪ Protective skin activity	The skin was significantly hydrated after applying the extract-enriched moisturizer. After 2 and 4 weeks, skin hydration levels in the test area inside the mask increased by 11.51% and 15.14%, respectively. After 2 and 4 weeks, the test area inside the mask showed a reduction in hemoglobin levels, which is an indication of erythema, by 5.06% and 6.74%, respectively.	[156]
	*Potentilla discolor*	▪ Anticancer activity	Significantly suppressed cell growth and induced apoptosis in both MC3 and YD15 cells. Produced upregulation of PUMA (p53 upregulated modulator of apoptosis) expression. The STAT3/PUMA axis was proven to be involved in extract-induced apoptosis after overexpression of STAT3, which partially restored cell growth inhibited by methanolic extract.	[157]
	*Potentilla tormentilla*	▪ Anti-inflammatory activity	The maximum inhibition of paw edema was achieved by 39.62% after carrageenan injection. The anti-inflammatory potential of PEG topical application was markedly dependent on time, as indicated by different percentages of inhibition during the follow-up period. Ellagic acid (the most prevalent bioactive compound in the extract) is thought to have inhibited inflammatory responses through p38 Mitogen-Activated Protein Kinase (MAPK) and signal transducers and activators of transcription (STAT) pathways. In addition, ellagic acid had a potent anti-inflammation effect against inflammation caused by carrageenan.	[158]
*Rosa*	*Rosa* *damascena*	▪ Anti-UVB-induced skin aging	Suppressed the activation of activator protein-1 (AP-1), leading to decreased transcription of matrix metalloproteinases. The extract reversed the UV-induced decrease in type 1 procollagen and TGF-β1 expression by modulating the transforming growth factor-beta (TGF-β) signaling pathway. Stimulated the TGF-β1-mediated Smad signaling pathway, involving Smad2/3 and Smad7, essential for transmitting TGF-β signals that regulate collagen production and degradation.	[159]
	*Rosa rugosa*	▪ Antioxidant, anti-inflammatory, anticancer activities	Suppressed melanin secretion and tyrosinase activity. It downregulated the expression of melanogenesis-related genes and proteins, including tyrosinase, MITF, TRP-1, and TRP-2, and inhibited the phosphorylation of PKA/CREB proteins.	[160]
*Crataegus*	*Crataegus pinnatifida* Bge.	▪ Anti-inflammatory, antioxidant, antimicrobial, and skin regeneration booster	LAG3, an inhibitory molecule that suppresses the activity of inflammatory T cells, was significantly increased, thus being responsible for the anti-inflammatory effect in rat skin. slpi, an antibacterial gene, was upregulated by aqueous leaf extract, exerting an anti-infective effect by stimulating antibacterial capacity in a group of rats. Thioredoxin (txn1) suppresses oxidative stress by regulating the dithiol/disulfide balance. Npas2 inhibition accelerates wound healing by increasing dermal collagen synthesis and collagen fiber formation.	[161]
	*Crataegus laciniata*	▪ Antimelanogenesis	The potential mechanism involved in the skin hyperpigmentation of flower extracts mediated by the inhibition of both monophenolase and diphenolase activities at low concentrations.	[162]
*Geum*	*Geum urbanum*	▪ Antioxidant, antiaging activity	Aqueous methanol extract of 80% methanol from the aerial parts of the plant and fractions obtained by extraction with solvents of increasing polarity suggest significant antioxidant activity, particularly the ethyl acetate fraction (IC50 of 14.7 µg/mL,) The ethyl acetate fraction also shows remarkable hydroxyl radical scavenging activity and copper ion-reducing capacity similar to trolox, a well-known antioxidant standard. Considering the role of elastases in the process of extracellular matrix remodeling and aging, their inhibition suggests a potential to combat the signs of aging by protecting the skin structure and reducing skin degradation. The ethyl acetate fraction (EAF) of *Geum urbanum* extract showed the best inhibitory activity on elastase, with an IC50 of 6.0 µg/mL, similar to quercetin, a positive standard.	[163]
*Filipendula*	*Filipendula glaberrima*	▪ Antioxidant, antiatopic, and anti-inflammatory	A strong free radical scavenging capacity, positively correlated with the high content of polyphenols and flavonoids in the flowering plant stage, was suggested by the lower IC50 values in the ABTS (0.16 mg/mL) and DPPH (0.55 mg/mL) tests compared to the IC50 value of 0.1 mg/mL obtained for ascorbic acid. The antiatopic potential is suggested by the inhibition of TNF-a + IFN-y-induced cytokine and chemokine production in HaCaT cells, the main cells involved in inflammatory skin responses. The anti-inflammatory activity is manifested by a significant decrease in the levels of IL-6, IL-8, and MCP-1, indicating that *Filipendula glaberrina* extracts may modulate the skin immune response by reducing the secretion of these inflammatory mediators.	[164]
*Sorbus*	*Sorbus commixta* Hedl.	▪ Antimelanoma	The butanol fraction of the ethanolic extract of *Sorbus commixta* fruits demonstrated significant cytotoxic activity on SK-MEL-2 melanoma cells (but not on HDFa dermal fibroblasts), suggesting a selective mechanism on tumor cells. The cytotoxic mechanism appears to involve inhibition of signaling through the MEK/ERK pathway, a pathway involved in cell proliferation and tumor cell survival, and increased caspase-3 activity, a marker of apoptosis.	[165]
*Fragaria*	*Fragaria ananassa* Duch. *(strawberry)*	▪ Antiproliferative	Strawberry flavonoid extracts have a significant antiproliferative effect on B16-F10 melanoma cells, reducing cell proliferation without causing high cytotoxicity, the effect being correlated with a decrease in the intracellular content of SPD and SPM, markers of cell proliferation. Anthocyanin-rich strawberry fruit extracts also influence the activity of tissue transglutaminase (TG2), a marker of cell differentiation, suggesting not only inhibition of proliferation but also induction of melanoma cell differentiation. Proteomic analysis revealed a significant change in protein expression, with a downregulation of proteins involved in tumor progression and metabolism, suggesting an alteration in the metabolic processes of cancer cells.	[166]

3T3—mouse embryonic fibroblast cell line; A375—human melanoma cell line; A431—human epidermoid carcinoma cell line; ABTS—2,2′-azino-bis(3-ethylbenzothiazoline-6-sulfonic acid); AD—atopic dermatitis; AP-1—activator protein-1; BALB/c—inbred mouse strain; B16-F10—murine melanoma cell line; caspase-3—cysteine–aspartic acid protease 3; CCD-986sk—human dermal fibroblast cell line; CREB—response element-binding protein; DPPH—2,2-diphenyl-1-picrylhydrazyl; ERK—Extracellular Signal-Regulated Kinase; HaCaT—human keratinocyte cell line; HDFa—human dermal fibroblast cell line; HMC-1—human mast cell line-1, IC_50_—half-maximal inhibitory concentration; IFN-γ—interferon gamma; IgE—Immunoglobulin E; IL-1β—Interleukin-1 beta; IL-12—Interleukin-12; IL-4—Interleukin-4; IL-5—Interleukin-5; IL-6—Interleukin-6; IL-8—Interleukin-8; LAG3—lymphocyte-activation gene 3; MAPK—Mitogen-Activated Protein Kinase; MAPKs—Mitogen-Activated Protein Kinases; MCP-1—monocyte chemoattractant protein-1; MEK—Mitogen-Activated Protein Kinase; MITF—microphthalmia-associated transcription factor; MMP-1—matrix metalloproteinase-1; MMP-13—matrix metalloproteinase-13; MMP-8—matrix metalloproteinase-8; Nf-κB—Nuclear Factor Kappa-light-chain-enhancer of activated B cells; NHDFs—normal human dermal fibroblasts; Npas2—neuronal PAS domain protein 2; PKA—protein kinase A; PUMA—p53 upregulated modulator of apoptosis; ROS—reactive oxygen species; S. aureus—Staphylococcus aureus; S. epidermidis—Staphylococcus epidermidis; SK-MEL-2—human melanoma cell line; slpi—secretory leukocyte protease inhibitor; Smad—Suppressor of Mothers against Decapentaplegic; SPD—Spermidine; SPM—Spermine; STAT3—signal transducer and activator of transcription 3; TARC—thymus and activation-regulated chemokine; TG2—tissue transglutaminase; TGF-β1—transforming growth factor-beta 1; TNF-α—tumor necrosis factor-alpha; TRP-1—tyrosinase-related protein 1; TRP-2—tyrosinase-related protein 2; TSRP—total saponin extract from *Rubus parvifolius*; Txn1—Thioredoxin 1; UV-B—ultraviolet B radiation; YD15—human oral cancer cell line.

## Data Availability

Not applicable.

## Abbreviations

The following abbreviations are used in this manuscript:

A375	Human melanoma cell line
ABTS	2,2′-azino-bis(3-ethylbenzothiazoline-6-sulphonic acid)
AGEs	Advanced glycation end products
AgNPs	Silver nanoparticles
AKT	Protein Kinase B
α-MSH pathway	Alpha-melanocyte-stimulating hormone
AQP3	Aquaporin 3
AP	Apple pomace
APG	Angiosperm Phylogeny Group
AME	*Aronia melanocarpa* extract
B16	Tumor line
B16-F10	Murine melanoma cell line
BRAF	A serine–threonine kinase in the family of *RAF* kinases
CDKN2A	Cyclin-dependent kinase inhibitor 2A
CAT	Catalase (antioxidant enzyme)
COX-1	Cyclooxygenase 1
COX-2	Cyclooxygenase 2
CPDs	Cyclobutane pyrimidine dimers
DOPA	3,4-dihydroxyphenylalanine
DMBA	7,12-dimethylbenz[a]anthracene
DNA	Deoxyribonucleic acid
DPPH	2,2-diphenyl-1-picrylhydrazyl
EBV-EA	Epstein–Barr virus early antigen
EC	Epicatechin
ERK	Extracellular Signal-Regulated Kinase
FcεRI	The high-affinity Fc receptor for immunoglobulin E
FGF-2	Fibroblast Growth Factor 2
FLG	Filaggrin
FRAP	Fluorescence recovery after photobleaching
GBA	Glucosylceramidase Beta
GCS	Glucosylceramide Synthase
HaCaT	Human keratinocyte cell line
H_2_O_2_	Hydrogen peroxide
HDFa	Human dermal fibroblasts
Hs27	Human Fibroblast Cell Line
HPLC-ESI-MS	High-performance liquid chromatography–electrospray ionization–mass spectrometry
IC50	Half-maximal inhibitory concentration
IgE	Immunoglobulin E
IFN-γ	Interferon gamma
IL-1β	Interleukin 1 beta
IL-10	Interleukin 10
IL-13	Interleukin 13
IL-17	Interleukin 17
IL-22	Interleukin 22
IL-23	Interleukin 23
IL-33	Interleukin 33
IL-36	Interleukin 36
IL-4	Interleukin 4
IL-5	Interleukin 5
IL-6	Interleukin 6
iNOS	Inducible Nitric Oxide Synthase
INV	Involucrin (structural protein in skin)
JNK	C-Jun N-Terminal Kinases
LC-MS	Liquid chromatography–mass spectrometry
L-DOPA	L-3,4-dihydroxyphenylalanine
LOX	Lipoxygenase
MAPK	Mitogen-Activated Protein Kinase
MMP-2	Matrix metalloproteinase 2
MMP-3	Matrix metalloproteinase 3
MMP-9	matrix metalloproteinase 9
MMPs	Matrix metalloproteinases
NHDFs	Normal human dermal fibroblasts
NO	Nitric oxide
NRAS	Neuroblastoma RAS Viral Oncogene Homolog
NC/Nga	Mouse models
p38 MAPK	p38 Mitogen-Activated Protein Kinases
PPARα, β/δ, and γ	Peroxisome Proliferator-Activated Receptors
PTEN	Phosphatase and tensin homolog on chromosome 10
PVDE	*Pourthiaea villosa* extract
TNF-α	Tumor necrosis factor-alpha
RCLE	*Rubus idaeus* lipid-soluble extract
REA	*Rosa multiflora* Thunb flowers and its subfractions in ethylacetate
RBT	*Rosa multiflora* Thunb flowers and its subfractions in n-butanol
RFCS	Rose flower cell sap
